# Intestinal organoids to model *Salmonella* infection and its impact on progenitors

**DOI:** 10.1038/s41598-024-65485-4

**Published:** 2024-07-02

**Authors:** Jin Yan, Claire Racaud-Sultan, Tiffany Pezier, Anissa Edir, Corinne Rolland, Coralie Claverie, Julien Burlaud-Gaillard, Michel Olivier, Philippe Velge, Sonia Lacroix-Lamandé, Nathalie Vergnolle, Agnès Wiedemann

**Affiliations:** 1 IRSD - Institut de Recherche en Santé Digestive, Université de Toulouse, INSERM, INRAE, ENVT, UPS, Toulouse, France; 2 Department of Gastroenterology, The Second Xiangya Hospital of Central South University, Changsha, China; 3 Research Center of Digestive Disease, Central South University, Changsha, China; 4 ISP, INRAE, Université de Tours, 37380 Nouzilly, France; 5 Plateforme IBISA de Microscopie Electronique, Université de Tours, CHRU de Tours, Tours, France

**Keywords:** *Salmonella*, Caecum, Stem cell, Progenitor, Organoid, EGFR, Biological techniques, Microbiology, Stem cells

## Abstract

In order to survive and replicate, *Salmonella* has evolved mechanisms to gain access to intestinal epithelial cells of the crypt. However, the impact of *Salmonella* Typhimurium on stem cells and progenitors, which are responsible for the ability of the intestinal epithelium to renew and protect itself, remains unclear. Given that intestinal organoids growth is sustained by stem cells and progenitors activity, we have used this model to document the effects of *Salmonella* Typhimurium infection on epithelial proliferation and differentiation, and compared it to an in vivo model of *Salmonella* infection in mice. Among gut segments, the caecum was preferentially targeted by *Salmonella*. Analysis of infected crypts and organoids demonstrated increased length and size, respectively. mRNA transcription profiles of infected crypts and organoids pointed to upregulated EGFR-dependent signals, associated with a decrease in secretory cell lineage differentiation. To conclude, we show that organoids are suited to mimic the impact of *Salmonella* on stem cells and progenitors cells, carrying a great potential to drastically reduce the use of animals for scientific studies on that topic. In both models, the EGFR pathway, crucial to stem cells and progenitors proliferation and differentiation, is dysregulated by *Salmonella,* suggesting that repeated infections might have consequences on crypt integrity and further oncogenesis.

## Introduction

*Salmonella* Typhimurium is a Gram-negative bacterium, infecting both humans and animals and responsible for a broad spectrum of diseases such as gastroenteritis, systemic infection, and asymptomatic carrier state according to the host. After oral contamination, the initiating step for *S.* Typhimurium infection requires its interaction with intestinal epithelium, which is the first host cellular barrier. As such, cells undergo perpetual cycles of proliferation and differentiation, driven by the regenerative activity of stem cells and progenitors^1^. Bacterial virulence factors and toxins hijack the mechanisms of epithelial cell regulation to promote host invasion and colonization and repeated infections are known to increase the risk of cancer secondary to tumor transformation of stem cells and progenitors^2^.

To face infection, epithelial secretory cells such as goblet and Paneth cells play important roles through the production of mucus and anti-microbial peptides, respectively^3^. Moreover, stem cells and progenitors are required to set a rapid and efficient repair following epithelial damage and cell death. Wnt and EGF ligands are critical to regulate signaling pathways such as β-catenin at the crossroad of stem cells and progenitors proliferation and differentiation^4^. In response to *S.* Typhimurium infection in streptomycin-pretreated mice, the proliferation of small intestinal epithelial cells and their β-catenin pathway are upregulated^5^, reflecting an increased turnover rate of small-intestinal epithelium. However, streptomycin antibiotic pretreatment disrupts intestinal microbiota, rendering mice more sensitive to *Salmonella* infection^6^, affecting both epithelial and immune functions^7–10^. Thus, a better knowledge of the impact of *S.* Typhimurium on stem cells and progenitors of the epithelial crypt in a physiological context is necessary.

In the last decade, methods to culture in vitro epithelial stem cells and progenitors, so-called organoids, have allowed to recapitulate specific interactions between microbes and epithelia^11^. Here, our objective was to compare a well-known model of in vivo* S.* Typhimurium infection in mice^12^ with an *S.* Typhimurium-infected organoid model that we have established. We have first determined that caecum was the gut segment preferentially targeted by *S.* Typhimurium in systemic infection mouse model, and we have studied the impact on caecal stem cells and progenitors of in vivo and in vitro* S.* Typhimurium infection.

## Results

### *S.* Typhimurium preferentially targets the caecal crypts

**Figure 1:**
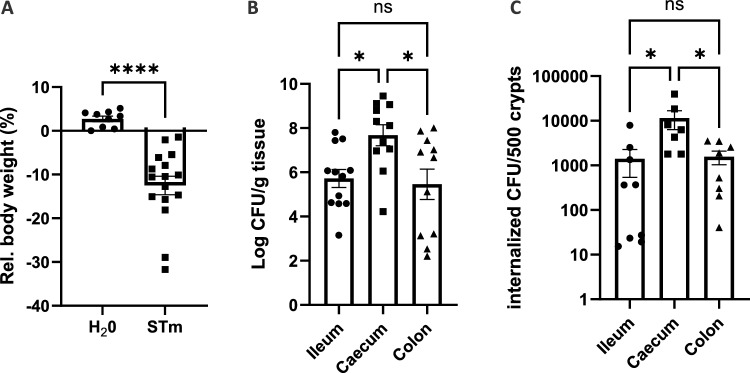
Caecal crypts preferentially targeted by *S.* Typhimurium. C57BL/6 mice were gavaged with water (H_2_O) or 2 × 10^8^ *S.* Typhimurium GFP (STm). (**A**) Relative body weight of the mice inoculated with *S.* Typhimurium GFP between day 0 and 4 p.i.. (**B**) Colonization of ileum, caecum, and colon 4 days p.i.. Enumeration was performed at 4 days p.i. after mouse killing as described in Materials and Methods. (**C**) CFU number of *S.* Typhimurium GFP per 500 purified crypts at 4 days p.i. after gentamycin treatment and crypt purification as described in Materials and Methods. The results are expressed as the mean of the relative value ± SEM from 3 independent experiments (at least 3 mice per experiment) (ns non-significant, *p < 0.05, ****p < 0.0001).

Susceptible mouse lineages (e.g., C57BL/6) are widely used to investigate the pathogenesis of *S.* Typhimurium infections. During the acute phase of infection, these animals develop overt signs of illness (including hunched posture, reduced movement, loss of body weight) typically appearing between 4 and 6 days post oral infection, with mortality occuring within the subsequent days. However, diarrhea does not develop^13–15^. To determine which intestinal segment is preferentially targeted by *S.* Typhimurium in mice, we determined the level of *S.* Typhimurium colonization in ileum, caecum, and colon of infected C57BL/6 mice at 4 days post-infection (p.i.). Typically, at 4 days p.i., symptoms start to be visible and a significant body weight loss occurs in mice infected with *S.* Typhimurium compared to controls (Fig. 1A). Then, the mice were sacrificed and the bacterial load was analyzed in whole tissues from the ileum, caecum, and colon. As shown in Fig. 1B, *S*. Typhimurium has the ability to colonize the different segments of the gut. However, the caecum was the most heavily colonized segment with approximately 10^7^ CFU/g tissue. Next, we analyzed the distribution of intracellular *S.* Typhimurium in intestinal-purified crypts. We have noticed that *S.* Typhimurium colonizes intestinal crypts with a similar profile to the whole tissue (Fig. 1C). Because the caecum was preferentially targeted by *S.* Typhimurium, caecal crypt was studied in the following experiments.

### *S*. Typhimurium enhances epithelial proliferation in caecal crypts

**Figure 2:**
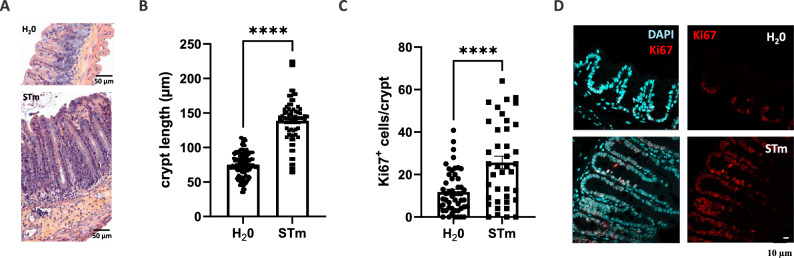
Caecal cell proliferation enhanced by *S.* Typhimurium C57BL/6 mice were gavaged with water (H_2_O) or 2 × 10^8^ *S.* Typhimurium GFP (STm). (**A**) A representative mouse histology section showing caecal tissue with hematoxylin and eosin stained at 4 days p.i.. Scale bars = 50 μm. (**B**) The depth of crypts was measured in 7 mice of 2 independent experiments with at least 10 crypts/mouse. The data show the mean ± SEM. (**C**) The graph shows the percentage of Ki67 positive cells relative to the total cells in cecal crypts in 9 mice of at least 2 independent experiments with at least 10 crypts/mouse. Values are means ± SEM. ****p < 0.0001. (**D**) Representative immunofluorescence staining of Ki67 in caecal crypts at 4 days p.i. Caecal tissue was processed for immunofluorescence and analyzed by confocal laser scanning microscopy. Horizontal sections of tissues are shown with nuclei (DAPI) in blue and proliferative cells (Ki67) in red. Scale bars = 10 μm.

As shown above, *Salmonella* has access to epithelial crypts, which contain stem cells and progenitors, responsible for the ability of the epithelium to renew and repair itself. To investigate the impact of *S.* Typhimurium on the proliferation area including stem cells and progenitors, the crypt length of mice was first evaluated at 4 days p.i.. H/E staining was used to quantify the caecal crypt length. The result revealed that the length of caecal crypts in the infected mouse group is significantly increased compared with the control group (Figs. 2A and B). In addition, immunofluorescence staining of Ki67, a marker of proliferative cells, indicated that the percentage of Ki67 positive cells per crypt is increased in the infected mouse group compared with the control group (Figs. 2C and D). Taken together, these data showed that *S.* Typhimurium infection results in enhanced stem cells and progenitors proliferation in the caecal crypt.

### *S.* Typhimurium impacts the progenitors functions through the EGFR pathway in caecal crypts

**Figure 3:**
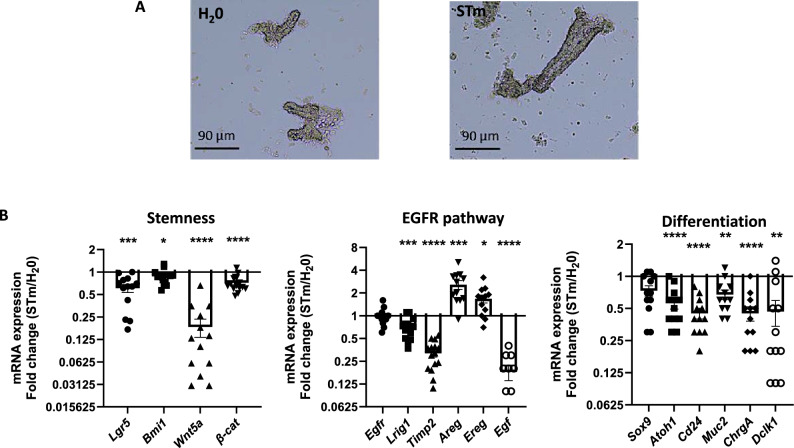
Modulation of caecal crypt proliferation by *S.* Typhimurium dependent on EGFR pathway. C57BL/6 mice were gavaged with water (H_2_O) or 2 × 10^8^ *S.* Typhimurium GFP (STm). 4 days p.i., the caecal crypts were purified (**A**) Representative images showing purified caecal crypts from control mice (H_2_0) and infected mice (STm) at 4 days p.i.. (**B**) Relative expression of specific genes involved in stem cells identity/proliferation (Stemness), stem cell and progenitor proliferation/migration (EGFR pathway), and progenitor differentiation (Differentiation) was determined in purified cecal crypts at 4 days p.i. of 2 independent experiments (at least 5 mice per experiment). Gene expression was normalized to the reference gene *hprt* and *gapdh*. Values are means ± SEM. *P < 0.05, **P < 0.01, ***P < 0.001, ****p < 0.0001.

In the crypt, the proliferation of stem cells and progenitors is mainly supported by Wnt- and EGF-dependent regulation. In purified caecal crypts (Fig. 3A), a study of mRNA expression (Fig. 3B) showed that, compared to control, *S.* Typhimurium infection induces a decrease of markers governing stem cell proliferation and identity (*Lgr5*, *Bmi1, β-catenin*) as well as *Wnt5a*, a Wnt ligand which has been shown to control the role of stem cells in epithelial repair^16^. By contrast, *S.* Typhimurium infection of caecal crypts modified the mRNA expression in favor of an EGFR-dependent regulation of cell proliferation (Fig. 3B), without modification of *Egfr* expression but with an increase of its ligands (*Areg*, *Ereg*, except *Egf*) and a decrease of its inhibitors (*Lrig1*, *Timp2*). Further, compared to control crypts, *S.* Typhimurium infection suggested a decrease of markers of the secretory pathway of differentiation (*Sox9, Atoh1*, *Cd24*, *ChgA*, *Dclk1*, *Muc2*) (Fig. 3B). The reduction of goblet cells/mucus in the caecal crypts has been verified by blue alcian staining (Figure S1).

Altogether, these data indicate a notable rise in proliferative cells (Ki67 positive cells per crypt) during *S.* Typhimurium infection. Within the crypt, proliferative cells encompass both stem cells and progenitors. Given the significant decrease in specific stem cell gene expression (Lgr5 and Bmi1) observed during *S.* Typhimurium infection, the heightened presence of proliferative cells may denote an increase in progenitors. This suggests that in the caecal crypt, progenitors but not stem cells are either direct or indirect affected by *S.* Typhimurium infection leading to excessive cell proliferation through an EGFR-dependent pathway.

### Setting up caecal organoid cultures for mimicking *S.* Typhimurium in vivo infection

**Figure 4:**
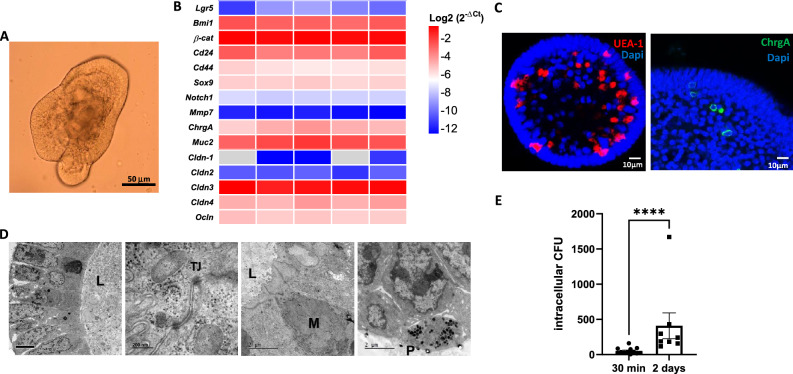
A caecal organoid model to mimic in vitro* S.* Typhimurium infection. After isolation from C57BL/6 mice, caecal crypts were cultured as organoids in 3D (Matrigel^™^) with L-WRN-conditioned medium as described in Materials and Methods. (**A**) Representative image of mature caecal organoid derived from C57BL/6 mouse after 7 days of culture in Matrigel^™^ with L-WRN medium. (**B**) Heat maps show the Log2^−ΔCt^ of a range of epithelial cell genes and colors represent scaled values of gene expression with blue for low and red for high values (**C**) Confocal laser scanning microscopy, showing secretory cells (UEA-1) in red, enteroendocrine cells (ChrgA) in green and nuclei (DAPI) in blue in mature caecal organoids. (**D**) Transmission electron microscopy images of mature caecal organoids: M, goblet cells, P, Paneth cells, L, lumen, TJ, tight junction. (**E**) Dissociated caecal organoid cells were infected with *S.* Typhimurium (about 3 × 10^5^ CFU/300 organoids) and then, cultured to form organoids as above but in the absence of exogenously added EGF. The number of internalized bacteria was quantified before being embedded in Matrigel^™^ (30 min) and after 2 days of culture (2 days p.i.). Values are means ± SEM of at least two independent experiments with 4 infected wells per condition. (****p < 0.0001).

Organoids are useful to study stem cells and progenitors functions and we aimed to establish caecal organoids for modeling *S.* Typhimurium infection, in order to mimic the bacterial impact observed in vivo. After their isolation from uninfected C57BL/6 mice, caecal crypts were cultured in 3D with L-WRN-conditioned medium, as organoids (Fig. 4A). In these culture conditions, we have verified by qRT-PCR the presence of the different cell types and barrier components in mature caecal organoids that normally constitute the mouse caecal epithelium in vivo. Markers of stem cells (*Lgr5*, *Cd44*, *Bmi1*, *β-catenin*), as well as markers of absorptive and secretory cell lineage differentiation (*Cd24*, *Sox9*, *Notch1, Muc2, ChrgA*) or tight junctions (*Cldn1*, *Cldn2*, *Cldn3*, *Cldn4*, *Ocln*), were all expressed in the caecal organoid cultures after 7 days of culture (Fig. 4B). Immunofluorescence staining (Fig. 4C) and transmission electron microscopy (TEM) (Fig. 4D) confirmed the presence and spatial organization of differentiated cells in mature caecal organoids. Ulex europaeous lectin and Chromogranin A staining demonstrated the presence of secretory and enteroendocrine cells, respectively (Fig. 4C). In mature organoids, TEM revealed a highly polarized organization of enterocytes with their typical apical brush border and cell-to-cell interactions such as tight junctions (Fig. 4D). Mature caecal organoids thus have basal-out conformation with the apical side facing the lumen of the organoid (Fig. 4D). Moreover, the presence of goblet cells with intracellular mucin granules is clearly distinguished, as well as the small black granules characteristics of Paneth cells (Fig. 4D).

Caecal organoids cultures were generated and then dissociated, to be co-incubated with *S.* Typhimurium. This cell suspension was subsequently cultured to allow the formation of new organoids. Importantly, and different from the above culture conditions, EGF was not added to the culture medium to preserve bacterial hijack on the EGFR pathway. Of note, while the starvation of EGF has contributed to the decrease in cell proliferation in uninfected organoids (Figures S2 A-C), the expression of epithelial genes displaced by *S.* Typhimurium infection in vivo was not significant (EGFR pathway, differentiation), except for enteroendocrine differentiation (CD24, ChgrA) which is increased as expected under low rate of progenitor proliferation^17^ (Figure S2D). Under these conditions, *S.* Typhimurium were able to enter epithelial cells and to replicate (Fig. 4E).

We have thus set up a caecal organoid model allowing in vitro infection of the epithelial crypt by *S.* Typhimurium.

### Caecal organoids infected in vitro recapitulate the impact of *S.* Typhimurium infection in vivo.

**Figure 5:**
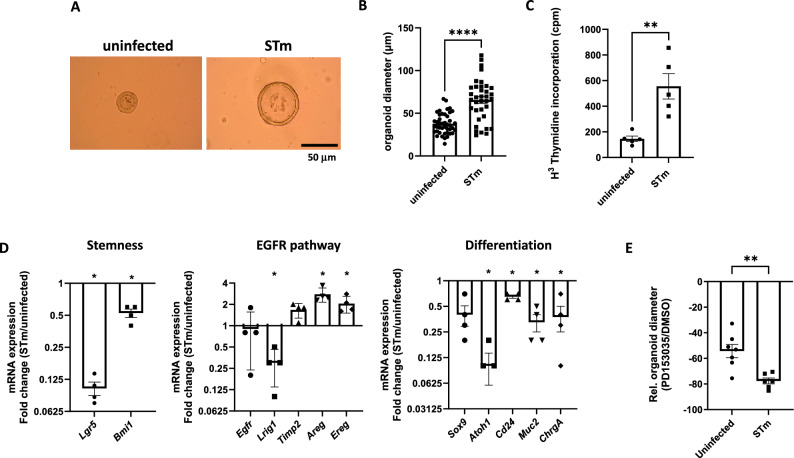
Caecal stem cells and progenitors functions controlled by *S.* Typhimurium in organoid in vitro model. Dissociated C57BL/6 caecal organoid cells were infected (STm; about 3 × 10^5^ CFU/300 organoids) or not (uninfected) and then cultured to form organoids in the EGF-depleted L-WRN medium. **(A**) Representative images of organoids 2 days p.i.. (**B**) Measurement of organoid diameter at 2 days p.i.. Values are means of 4 wells ± SEM of a representative experiment of two independent trials with at least 10 organoids/well (**C**) Incorporation of H^3^-Thymidine in caecal organoids (counts per minute: cpm) at 2 days p.i.. (**D**) Relative gene expression of specific genes involved in intestinal cell proliferation/differentiation in infected (STm) and uninfected caecal organoids. Results are expressed as mean ± SEM of 2^−ΔCt^ (fold change) between infected (STm) and uninfected conditions. (**E**) Uninfected and (STm) infected dissociated organoids were cultured in the medium containing EGFR inhinitor (PD153035, 1 µM) or its solvent (DMSO) as control. The organoid diameter was measured at 6 days p.i. to calculate the relative organoid diameter in infected (STm) and uninfected caecal organoids between PD153035 vs DMSO tretament. Values are means of at least 4 wells ± SEM of a representative experiment of two independent trials (*p < 0.05, **, p < 0.01, ****p < 0.0001).

We evaluated the impact of in vitro* S.* Typhimurium infection on caecal organoids and then compared it to our above results on in vivo infection of caecal crypts. Compared to controls, infected organoids were bigger (Figs. 5A and B) with a higher incorporation of H^3^-thymidine (Figs. 5C). Thus, as shown previously after infection of crypts in vivo, the stem cells and progenitors proliferation was enhanced after in vitro infection of organoids by *S.* Typhimurium. Moreover, as described above in infected crypts, markers of mRNA expression of stem cells and the secretory cell lineage pathway of differentiation decreased whereas those of the EGFR pathway increased in infected organoids, compared to controls (Fig. 5D). To confirm the involvement of the EGFR pathway, dissociated single cells derived from caecal organoids were infected with *S.* Typhimurium and cultured in the presence of an EGFR inhibitor, PD153035, or DMSO as control. After 6 days of culture, the growth of the organoids was estimated by measuring the organoid diameter. As shown in Fig. 5E, the inhibition of the EGFR pathway has an impact on organoid growth both in control and infected conditions. However, compared to controls, EGFR blockade has a stronger effect on caecal organoids infected with *S.* Typhimurium. Interestingly, the impact of EGFR inhibition on infected organoids compared to control organoids, is in the same range than overgrowth due to *S*. Typhimurium (Fig. 5B), suggesting that enhanced cell proliferation secondary to infection is mainly EGFR-dependent.

In conclusion, we demonstrated that in our culture conditions, the infection of caecal organoids with *S.* Typhimurium recapitulates the main effects on proliferation and differentiation of stem cells and progenitors measured upon in vivo infection, highlighting a major role of the EGFR pathway.

## Discussion

The comprehension of mechanisms supporting the impact of *S.* Typhimurium infection on stem cells and progenitors in the intestinal crypt is crucial to preventing hard consequences on the digestive tract. Also, the recent development of organoid technology prompts researchers to search for an in vitro model of infection capable of reproducing these changes in stem cells and progenitors functions. In this study, we have demonstrated that caecal organoids infected in vitro with *S.* Typhimurium display similar changes in stem cells and progenitors behavior compared to caecal crypts infected in vivo with *S.* Typhimurium: enhanced proliferation, diminished secretory differentiation, and promotion of the EGFR pathway.

First, we showed that the caecum, rather than the ileum and the colon, is preferentially colonized by *S*. Typhimurium in a systemic infection mouse model with endogenous intestinal microbiota. This finding has previously been observed in a streptomycin pre-treated mouse model containing a reduced intestinal microbiota as well as in chicken containing intestinal microbiota^18,19^. These data suggest that the presence/absence of microbiota does not seem to interfere with the intestinal segment preferentially colonized by *S.* Typhimurium. Further, our results definitively establish that the caecum is the preferred location of the gastrointestinal tract for *S.* Typhimurium colonization.

The next step in our study was to characterize the caecal crypt proliferative activity during *S.* Typhimurium infection. Our results reveal an increase in cell numbers in infected caecal crypts as previously shown in *S.* Typhimurium-infected crypts of the murine small intestine^5^. Increasing crypt cell proliferation has been previously observed as a consequence of intestinal infection by other enteropathogens such as *Erwinia carotovora* in *Drosophila*^20^, *Lawsoniain tracellularis* in pig^21^, and *L. monocytogenesis*^22^. These studies pointed out that intestinal cell proliferation could result from both bacterial control- and epithelial repair-based mechanisms.

Crypt cell proliferation is supported by stem cells and progenitors. According to the literature, in response to an excision in murine intestine, Wnt5a, a noncanonical Wnt ligand, is required for crypt regeneration, with its effects mediated by the activation of transforming growth factor-β (TGF-β) signaling^16^. Considering the presence of TGF-β inhibitor, A83-01, in our organoid culture medium, and the observed decrease in Wnt5a gene expression under infected conditions compared to uninfected conditions, we believe that the observed mechanism does not support this type of repair process. However, in conditions of acute injury and colitis, the plasticity of immature cells allows mechanisms of dedifferentiation, reserve stem cell activation and reprogramming giving activated stem cells and progenitors capable to regenerate at the end of the process all cell types of the crypt^23^. Early step of repair associates cell proliferation and decrease of secretory differentiation based on the properties of a subtype of progenitor cells^23^. Accordingly, our data show that *S.* Typhimurium infection triggers enhanced cell proliferation associated with decreased Lgr5 positive stem cells and secretory differentiation. So, both in our in vivo and in vitro models, epithelial progenitors, at the crossroad of stem cells and differentiated cells^24,25^, might play a critical role to face *Salmonella* infection.

*S.* Typhimurium may directly or indirectly hijack the intestinal progenitor proliferation by controlling EGFR signaling. Indeed, EGFR has been implicated in the balance between secretory and absorptive cell types in the crypt^26^. Here, our data show that *Egfr* expression is unchanged in both models of in vivo and in vitro* S.* Typhimurium infection. However, promotors of its activation (*Areg*, *Ereg*) are increased whereas its inhibitors (*Lrig1*, *Timp2*) are decreased. To investigate the potential involvement of EGFR signaling on the cell proliferation mechanism controlled by EGFR, we used an EGFR inhibitor exclusively in the organoid model because using an EGFR inhibitor in vivo is challenging due to its implication in several vital mechanisms. Our data indicate that EGFR activity is required for the overgrowth of *S*. Typhimurium-infected organoids compared to controls. Taken together, our data show that STm impacts EGFR activity. However, since *S.* Typhimurium targets EGFR to invade epithelial cells^27^, it is challenging to distinguish between EGFR signaling associated with bacterial invasion and that which controls cell proliferation. In the small intestine of *S*. Typhimurium infected mice pretreated with streptomycin, Liu et al. measured an increased turnover rate of the intestinal epithelium linked to the Wnt/β-catenin pathway^5^. Our different results can be explained by the different intestinal segments studied and the mouse model using or not streptomycin pre-treatment conditions. Indeed, streptomycin treatment is known to alter the microbiota and to induce many changes in the gastrointestinal tract such as a dramatically larger caecum, longer crypt, and increased intestinal cellular proliferation by activating the Wnt/ β-catenin signaling pathway^7^. As we obtained the same results in both infected caecal crypts and organoids, we may speculate that the presence of intestinal microbiota does not play a role in this mechanism.

The *Salmonella* virulence factor, driving to the progenitor cell proliferation by activating the EGFR pathway remains to be identified. One candidate may be the *Salmonella* Rck outer membrane protein, known to allow *S.* Typhimurium invasion, through the binding with the EGFR host receptor^27^. However, it has been shown that Rck delays the cell cycle of the infected intestinal cancer cell line HCT116^28^. In addition, it should be noted that Rck production is directly regulated by quorum sensing, which involves SdiA in an N-acylhomoserine lactone dependent manner^29,30^, suggesting that the Rck production by *Salmonella* requires intestinal microbiota presence. Thus, more studies are required to definitively identify the *Salmonella* virulence factor controlling caecal cell proliferation, and the signaling pathway targeted by the bacteria.

The epithelial overgrowth observed during *Salmonella* infection can be problematic. Indeed, excessive proliferation of intestinal crypts can lead to an altered intestinal structure, a decreased nutrient absorption, and an increased risk of tumor development. Thus, repeated *Salmonella* infections raise the question of their long-term consequences on the crypt integrity and its dysregulation in pathologies such as cancer. In 2015, Scanu et al*.* showed an impact of *S.* Typhimurium on Akt and MAP pathways to induce malignant transformation in fibroblasts, murine gallbladder organoids, and predisposed mice^31^. Thanks to the caecal organoid model that we established here, the identification of the pathway triggered by *S.* Typhimurium infection is currently being investigated in our lab. The organoid model carries great potential to investigate precisely the pathophysiology of the interaction of bacteria with the intestinal epithelium^32^ and to drastically reduce the number of animals used in the future. Particularly, our present work should help to develop experiments on *S.* Typhimurium infection using human organoids.

## Methods

### Bacterial strains and culture conditions

The strain *S.* Typhimurium 14028 (ATCC) was grown at 37 °C overnight with shaking at 150 rpm in Luria–Bertani broth (LB, Sigma Aldrich). GFP expressing strain used in this study was obtained by bacterial electroporation of plasmid pSUP202 GFP^28^.

### Animal experiments

All experiments were conducted according to the Guide for the Care and Use of Laboratory Animals of the European Council, the Animal Care. 8 week-old male C57BL/6 mice (Janvier Laboratories, Saint Quentin Fallavier, France) housed under specific-pathogen-free conditions, were orally gavaged with sterile H_2_0 or 2 × 10^8^ *S.* Typhimurium GFP in 100 µL water. At 4 days post-infection (p.i.), the mice were sacrificed and ileum, caecum, and colon samples were aseptically removed and the luminal content was discarded from the tissue. Then, the tissues were either (i) homogenized using Precellys^®^ 24 homogenizer (Bertin) in 700 µL LB and plated for CFU enumeration on LB agar plates supplemented with 100 µg/mL carbenicillin (Cb100; Sigma Aldrich); (ii) washed in PBS containing gentamicin at 100 µg/mL before crypt purification; (iii) fixed in formol and then embedded in paraffin.

### Histology, immunostaining, and confocal microscopy

5 µm paraffin-embedded sections were cut and de-waxed before staining. (i) Subsequent hematoxylin and eosin (H/E) and blue alcian staining was performed on the caecal sections. The images were captured with a slide scanning microscope (Pannoramic 250 FLASH III 3.0.0, 3DHISTECH). Ten intact and well-oriented with longitudinally cut crypts per mouse were measured. In all cases, at least ten mice per condition were analyzed as detailed in each figure legend. (ii) 5 µm paraffin-embedded sections were subjected to a heat-induced epitope retrieval step before immunostaining with Ki67. After rehydration, the slides were placed in 10 mM citrate buffer adjusted to pH 6.0 at 95 °C for 45 min. The slides were then rinsed with water. To avoid nonspecific staining, tissue sections were incubated for 90 min in saturation buffer (PBS containing 1% BSA (Dominique Dutscher), 0.5% triton X-100 (Sigma Aldrich) and 0,01% tween (Sigma Aldrich)). Overnight incubation with anti-Ki67 (diluted 1:200 in saturation buffer; Abcam ab16667), was carried out at 4 °C. After rinsing in PBS containing tween 0,01%, the sections were incubated for 60 min at room temperature with a 1:1000 dilution of donkey anti-rabbit IgG Alexa Fluor 555 (Invitrogen) in saturation buffer. After rinsing in PBS containing tween 0,01%, sections were finally mounted with Prolong Gold antifade reagent with DAPI containing ProLong medium (Thermo Fisher Scientific). Tissue was imaged using laser-scanning confocal microscope LSM710 (Zeiss).

### Crypt purification

The ileum, caecum, and colon were removed and placed in phosphate-buffered saline without Ca^2+^ and Mg^2+^ (PBS; Thermo Fisher Scientific). The crypts were isolated as described by Berger et al*.*^33^. The number of crypts was estimated using a bright field optical microscopy (Nikon). About 1000 crypts were embedded in 50 μL Matrigel^™^ (Corning). After 20 min at 37 °C in a humidified atmosphere at 5% CO_2_, L-WRN medium (DMEM/F-12 Glutamax-Hepes (Gibco Life Technologies, Paisley, UK) supplemented with 50% L-WRN supernatant (ATCC^®^ , CRL-3276, Lacroix-Lamande et al.^34^), 50 ng/mL mouse EGF (Sigma Aldrich), B27 1X (ThermoFisher Scientific), 10 µM SB2022190 (Tocris),500 nM A83-01 (Tocris), 1 mM *N*-acetyl-L-cysteine (Sigma Aldrich), 10 nM gastrin I (Tocris) and 10 µM Y27632 (Tocris) were added and incubated at 37 °C with 5% L-WRN medium was changed every 2–3 days.

### qRT-PCR

Total RNAs from caecal crypts or organoids were extracted using TRI reagent^®^ (Euromedex). Samples were purified and treated DNAse using the Direct-zol RNA kit (Zymo Research—Ozyme) according to the manufacturer’s instructions. RNAs were quantifed by the absorbance A_260_ (Nanodrop, NanoPhotometer^®^ P-330 Implen, Thermo Fisher Scientific) and reverse transcript using the Maxima first strand kit (Fermentas, Thermo Fisher Scientific). Quantitative PCR was performed on a LightCycler 480 Instrument (Roche) with 45 ng cDNA, Takyon^™^ NO ROX SYBR Mmx dTTP blue (Eurogentec), and 0.6 µM gene primers (amplification 40 cycles, 60 °C). *Hprt*, *Gapdh*, and *TBP* genes were used as housekeeping genes, and the primer sequences used in this study are listed in Table 1. The gene expression level was normalized to values obtained from housekeeping genes. Fold change between mRNA levels of H_2_O/uninfected samples versus infected with S. Typhimurium pSUP202 GFP was calculated with the comparative 2^−ΔΔCt^ method.

**Table 1:** Mouse sequence primers used in this study.

Mouse target gene	Forward sequence 5'-3'	Reverse sequence 5'-3'
Areg	GCTGAGGACAATGCAGGGTAA	AGTGACAACTGGGCATCTGG
Atoh1	GCTTATCCCCTTCGTTGAA	TCTTTTACCTCAGCCCAC
B-catenin	GCTATTCCACGACTAGTTCAG	GGAATGGTATTGAGTCCTCG
Bmi1	TCCCCACTTAATGTGTGTCCT	CTTGCTGGTCTCCAAGTAACG
Cd24	GGCACTGCTCCTACCCACGC	CACCCCCTCTGGTGGTAGCG
Cd44	TCTGCCATCTAGCACTAAGAGC	GTCTGGGTATTGAAAGGTGTAGC
ChgA	TCCCCACTGCAGCATCCAGTTC	CCTTCAGACGGCAGAGCTTCGG
Cldn1	CCTACTTTCCTGCTCCTG	TGTCCATTTTGTATTTGCTCC
Cldn2	CCCACAGATACTTGTAAGGAG	CCAAAAGGCCTAGGATGTAG
Cldn3	ACTGCGTACAAGACGAGACG	GGGCACCAACGGGTTATAGA
Cldn4	CCACTCTGTCCACATTGCCT	CTTTGCACAGTCCGGGTTTG
Dclk1	CTGCAGCAGGAGTTTCTGTA	CCGAGTTCAATTCCGGTGGA
Egf	AAGGATCCTGACCCCGAACT	TGGGGCATGTGCAGTGATAG
Egfr	GCTGAGAAAGACTGCAAGGCC	CAGCCTTCCGAGGAGCATAA
Ereg	TGACGCTGCTTTGTCTAGGTTC	ATGCATGATGGGATCACGGT
Lgr5	CTACTCGAAGACTTACCCAGT	GCATTGGGGTGAATGATAG
Lrig1	ACAATCGAGGATACCAGTG	TCCAAGGTTCAGGTGTTC
Mmp7	GCAGGCATTCAGAAGTTATATG	ACAAGGAAGAGGGAAACAG
Muc2	GTAAACTGCTCTCTGGACTG	CTTGGAAGACGTGGTAGATG
Notch1	ACATCCGTGGCTCCATTGTCTA	GACGCAAGAGCACCTAGGAAGG
Ocln	ACCCTGACCACTATGAAAC	CGTCTAGTTCTGCCTGTAAG
Sox9	GAGCCGGATCTGAAGAGGGA	GCTTGACGTGTGGCTTGTTC
Timp2	CAACAGGCGTTTTGCAATGC	ATCCTCTTGATGGGGTTGCC
Wnt5a	GTCCTTTGAGATGGGTGGTATC	ACCTCTGGGTTAGGGAGTGTCT
Gapdh	AGGTCGGTGTGAACGGATTTG	TGTAGACCATGTAGTTGAGGTCA
Hprt	TCAGTCAACGGGGGACATAAA	GGGGCTGTACTGCTTAACCAG
TBP	CAGCCTTCCACCTTATGCTC	TTGCTGCTGCTGTCTTTGTT

### Transmission electron microscopy

Organoids were collected and postfixed for 1 h with 2% osmium tetroxide (Agar Scientific) as previously described in Lacroix-Lamandé et al.^34^. Briefly, organoids were dehydrated in a graded series of ethanol solutions and propylene oxide and then embedded in pure resin (Sigma Aldrich). 90 nm organoid-embedded sections were obtained with a Leica EM UC7 ultramicrotome (Leica Microsystems) and then stained with 5% uranyl acetate (Agar Scientific), and 5% lead citrate (Sigma Aldrich). Examinations were made with a JEOL 1011 transmission electron microscope.

### *S*. Typhimurium infection of organoids

3D caecal organoids were dissociated into single cells in TrypLE^™^ (Gibco Life Technologies) according to manufacturer recommendations. Depending on the number of organoids, the single cells were incubated with either DMEM/F-12 Glutamax-Hepes or DMEM/F-12 Glutamax-Hepes containing *S.* Typhimurium GFP (about 3 × 10^5^ CFU/300 organoids) for 30 min at 37 °C. After 3 washes with DMEM/F-12 Glutamax-Hepes containing 100 µg/mL gentamicin, the single cells were embedded in Matrigel^™^ and then either L-WRN medium without EGF containing 10 µg/mL gentamicin to reform organoids. To inhibit the EGFR pathway, PD153035 (1 µM) or DMSO as control, was added to DMEM containing or not *S.* Typhimurium and in L-WRN medium to reform organoids. The organoids were incubated at 37 °C at 5% CO_2_ in a humidified atmosphere. Images were captured with a bright field optical microscopy (Nikon). Diameter of ten organoids per well were measured and the relative organoid diameter has been calculated as follow: Rel. organoid diameter = [(organoid diameter of treated sample—mean of untreated organoid diameter) * 100]/mean of untreated organoid diameter.

### H^3^-Thymidine incorporation assay

H^3^-thymidine at 1 mCi/3.76 × 104 Bq (PerkinElmer) was added to uninfected and infected dissociated cells embedded in Matrigel™, followed by scintillation counting (Packard 1600 TR meter, Meriden, CT)^28^.

### Statistical analysis

To analyze the statistical differences between two groups, a Mann–Whitney test was used with a low number of samples and an unpaired t-test when the number of samples exceeded 30. Differences among three groups were analyzed using ANOVA (Prism, version 6.0; GraphPad Software, La Jolla, CA, USA). P-values of 0.05 or less were statistically considered significant.

### Ethics approval and consent to participate

All experiments were approved by Ethics Committee of US006/CREFE (CEEA-122; application number APAFIS no. 22-U1220-NV/AW-014). The institutional guidelines are in compliance with the ARRIVE guidelines.

### Supplementary Information

Supplementary Legends.

Supplementary Figures.

## Data Availability

All data generated or analyzed during this study are included in this published article [and its supplementary information files].

## References

[1] Gehart H, Clevers H (2019). Tales from the crypt: New insights into intestinal stem cells. Nat. Rev. Gastroenterol. Hepatol..

[2] Shanker EB, Sun J (2023). *Salmonella* infection acts as an environmental risk factor for human colon cancer. Cell Insight.

[3] Hou Q (2022). Bacillus subtilis programs the differentiation of intestinal secretory lineages to inhibit *Salmonella* infection. Cell Rep..

[4] Wang Z, Qu Y-J, Cui M (2023). Modulation of stem cell fate in intestinal homeostasis, injury and repair. World J. Stem Cells.

[5] Liu X, Lu R, Wu S, Sun J (2010). *Salmonella* regulation of intestinal stem cells through the Wnt/β-catenin pathway. FEBS Lett..

[6] Barthel M (2003). Pretreatment of mice with streptomycin provides a *Salmonella enterica* serovar Typhimurium colitis model that allows analysis of both pathogen and host. Infect. Immun..

[7] Kennedy EA, King KY, Baldridge MT (2018). Mouse microbiota models: Comparing germ-free mice and antibiotics treatment as tools for modifying gut bacteria. Front. Physiol..

[8] Reikvam DH (2011). Depletion of murine intestinal microbiota: Effects on gut mucosa and epithelial gene expression. PLoS One.

[9] Ekmekciu I, Fiebiger U, Stingl K, Bereswill S, Heimesaat MM (2017). Amelioration of intestinal and systemic sequelae of murine Campylobacter Jejuni infection by probiotic VSL#3 treatment. Gut Pathog..

[10] Wilen CB (2018). Tropism for tuft cells determines immune promotion of norovirus pathogenesis. Science.

[11] Puschhof J, Pleguezuelos-Manzano C, Clevers H (2021). Organoids and organs-on-chips: Insights into human gut-microbe interactions. Cell Host Microbe.

[12] Rossi O, Vlazaki M, Kanvatirth P, Restif O, Mastroeni P (2020). Within-host spatiotemporal dynamic of systemic salmonellosis: Ways to track infection, reaction to vaccination and antimicrobial treatment. J. Microbiol. Methods.

[13] Santos RL (2001). Animal models of *Salmonella* infections: Enteritis versus typhoid fever. Microbes Infect..

[14] Nilsson OR, Kari L, Steele-Mortimer O (2019). Foodborne infection of mice with *Salmonella* Typhimurium. PLoS One.

[15] Walker GT, Gerner RR, Nuccio S-P, Raffatellu M (2023). Murine models of *Salmonella* infection. Curr. Protoc..

[16] Miyoshi H, Ajima R, Luo CT, Yamaguchi TP, Stappenbeck TS (2012). Wnt5a potentiates TGF-β signaling to promote colonic crypt regeneration after tissue injury. Science.

[17] Basak O (2017). Induced quiescence of Lgr5+ stem cells in intestinal organoids enables differentiation of hormone-producing Enteroendocrine cells. Cell Stem Cell.

[18] Kyrova K (2014). The response of porcine monocyte derived macrophages and dendritic cells to *Salmonella* Typhimurium and lipopolysaccharide. BMC Vet. Res..

[19] Furter M, Sellin ME, Hansson GC, Hardt W-D (2019). Mucus architecture and near-surface swimming affect distinct *Salmonella* Typhimurium infection patterns along the murine intestinal tract. Cell Rep..

[20] Buchon N, Broderick NA, Kuraishi T, Lemaitre B (2010). Drosophila EGFR pathway coordinates stem cell proliferation and gut remodeling following infection. BMC Biol..

[21] Huan YW (2017). *Lawsonia intracellularis* exploits β-catenin/Wnt and Notch signalling pathways during infection of intestinal crypt to alter cell homeostasis and promote cell proliferation. PLoS One.

[22] Huang J, Zhou C, Zhou G, Li H, Ye K (2021). Effect of Listeria monocytogenes on intestinal stem cells in the co-culture model of small intestinal organoids. Microb. Pathog..

[23] Liu CY (2023). Wound-healing plasticity enables clonal expansion of founder progenitor cells in colitis. Dev. Cell.

[24] Yan KS (2017). Intestinal Enteroendocrine lineage cells possess homeostatic and injury-inducible stem cell activity. Cell Stem Cell.

[25] Jadhav U (2017). Dynamic reorganization of chromatin accessibility signatures during dedifferentiation of secretory precursors into Lgr5+ intestinal stem cells. Cell Stem Cell.

[26] Sanman LE (2021). Transit-amplifying cells coordinate changes in intestinal epithelial cell-type composition. Dev. Cell.

[27] Wiedemann A (2016). Identification of the epidermal growth factor receptor as the receptor for *Salmonella* Rck-dependent invasion. FASEB J..

[28] Mambu J (2020). Rck of *Salmonella* Typhimurium delays the host cell cycle to facilitate bacterial invasion. Front. Cell Infect. Microbiol..

[29] Abed N (2014). Direct regulation of the pefI-srgC operon encoding the Rck invasin by the quorum-sensing regulator SdiA in *Salmonella* Typhimurium. Mol. Microbiol..

[30] Smith JN, Ahmer BMM (2003). Detection of other microbial species by *Salmonella*: Expression of the SdiA regulon. J. Bacteriol..

[31] Scanu T (2015). *Salmonella* manipulation of host Signaling pathways provokes cellular transformation associated with gallbladder carcinoma. Cell Host Microbe.

[32] Aguirre Garcia M (2022). Intestinal organoids: New tools to comprehend the virulence of bacterial foodborne pathogens. Foods.

[33] Berger M (2022). Prenatal stress induces changes in PAR2- and M3-dependent regulation of colon primitive cells. Am. J. Physiol. Gastrointest. Liver Physiol..

[34] Lacroix-Lamandé S (2023). Differential *Salmonella* Typhimurium intracellular replication and host cell responses in caecal and ileal organoids derived from chicken. Vet. Res..

