# Supplementary material for: Intestinal organoids to model Salmonella infection and its impact on progenitors
Source: Sci Rep. 2024 Jul 2;14:15160. doi: 10.1038/s41598-024-65485-4 (PMC11219929; doi:10.1038/s41598-024-65485-4)
Supplement: Supplementary file 1 — Supplementary Legends. [file 41598_2024_65485_MOESM1_ESM.docx]

**Supplementary figure legends**

**Figure S1:** **Impact of *S.* Typhimurium on goblet cells**. C57BL/6 mice were gavaged with water (H_2_O) or 2x10^8^ *S.* Typhimurium GFP (STm). (**A**) A representative mouse histology section showing caecal tissue with blue alcian stained at 4 days p.i.. Scale bars = 50 μm. (**B**) The number of goblet positive cells per crypt was counted in at least 3 mice of 3 independent experiments. The data show the mean ± SEM. ****p<0.0001.

**Figure S2. Intestinal proliferation controlled by EGF in organoid *in vitro* model.** Dissociated C57BL/6 caecal organoid cells were cultured to form organoids in complete (+EGF) or EGF-depleted (-EGF) L-WRN medium. **(A)** Representative images of organoids 2 days p.i.. (**B**) Measurement of organoid diameter in µm (**C**) Incorporation of H^3^-Thymidine in caecal organoids (counts per minute: cpm) 2 days p.i.. (**D**) Relative gene expression of specific genes involved in intestinal cell proliferation/differentiation in the absence of EGF. Results are expressed as mean ± SEM of 2^-ΔCt^ (fold change) between +EGF and -EGF. (*p<0.05).
