# Supplementary figures and images for: Intestinal organoids to model Salmonella infection and its impact on progenitors
Source: Sci Rep. 2024 Jul 2;14:15160. doi: 10.1038/s41598-024-65485-4 (PMC11219929; doi:10.1038/s41598-024-65485-4)

## Slide 1
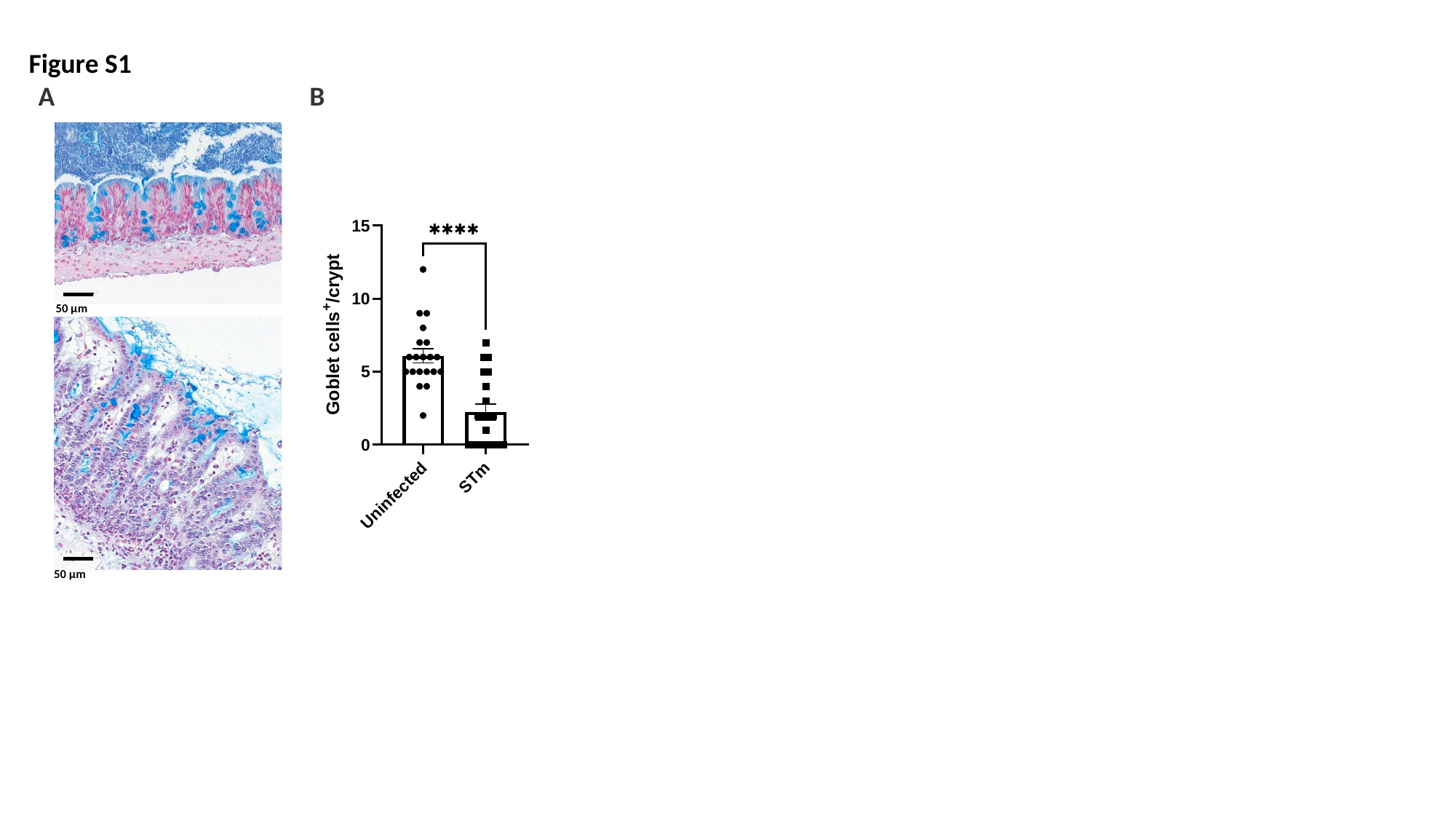

Figure S1
A
B
50 µm
50 µm

## Slide 2
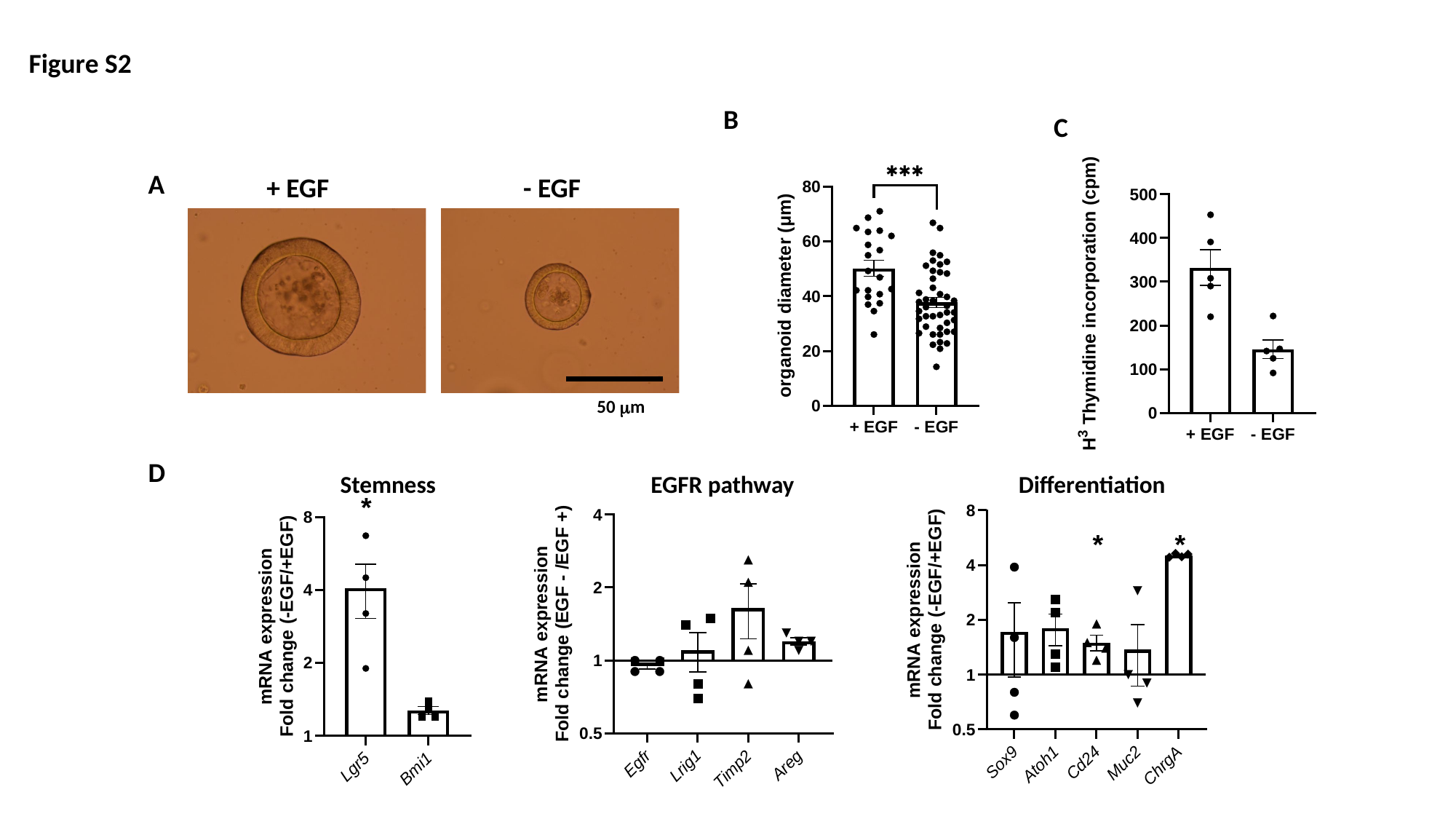

Figure S2
B
C
A
+ EGF
- EGF
50 mm
D
Stemness
EGFR pathway
Differentiation

Supplement: Supplementary file 2 — Supplementary Figures. [file 41598_2024_65485_MOESM2_ESM.pptx]
